# Magnetite nanoparticles: an emerging adjunctive tool for the improvement of cancer immunotherapy

**DOI:** 10.37349/etat.2024.00220

**Published:** 2024-04-23

**Authors:** Phoomipat Jungcharoen, Kunakorn Thivakorakot, Nachayada Thientanukij, Natkamon Kosachunhanun, Chayanittha Vichapattana, Jutatip Panaampon, Charupong Saengboonmee

**Affiliations:** Changchun Institute of Applied Chemistry, Chinese Academy of Sciences, China; ^1^Department of Environmental Engineering, Faculty of Engineering, Khon Kaen University, Khon Kaen 40002, Thailand; ^2^Cho-Kalaphruek Excellent Research Project for Medical Students, Faculty of Medicine, Khon Kaen University, Khon Kaen 40002, Thailand; ^3^Department of Biochemistry, Faculty of Medicine, Khon Kaen University, Khon Kaen 40002, Thailand; ^4^Division of Hematologic Neoplasia, Department of Medical Oncology, Dana-Farber Cancer Institute, Boston, MA 02215, USA; ^5^Department of Medicine, Harvard Medical School, Boston, MA 02215, USA; ^6^Division of Hematopoiesis, Joint Research Center for Human Retrovirus Infection, Kumamoto University, Kumamoto 8600811, Japan; ^7^Center for Translational Medicine, Faculty of Medicine, Khon Kaen University, Khon Kaen 40002, Thailand

**Keywords:** Cancer, magnetic iron oxide nanoparticles, immunotherapy, tumor microenvironment

## Abstract

Cancer immunotherapy has emerged as a groundbreaking field, offering promising and transformative tools for oncological research and treatment. However, it faces several limitations, including variations in cancer types, dependence on the tumor microenvironments (TMEs), immune cell exhaustion, and adverse reactions. Magnetic nanoparticles, particularly magnetite nanoparticles (MNPs), with established pharmacodynamics and pharmacokinetics for clinical use, hold great promise in this context and are now being explored for therapeutic aims. Numerous preclinical studies have illustrated their efficacy in enhancing immunotherapy through various strategies, such as modulating leukocyte functions, creating favorable TMEs for cytotoxic T lymphocytes, combining with monoclonal antibodies, and stimulating the immune response via magnetic hyperthermia (MHT) treatment (Front Immunol. 2021;12:701485. doi: 10.3389/fimmu.2021.701485). However, the current clinical trials of MNPs are mostly for diagnostic aims and as a tool for generating hyperthermia for tumor ablation. With concerns about the adverse effects of MNPs in the *in vivo* systems, clinical translation and clinical study of MNP-boosted immunotherapy remains limited. The lack of extensive clinical investigations poses a current barrier to patient application. Urgent efforts are needed to ascertain both the efficacy of MNP-enhanced immunotherapy and its safety profile in combination therapy. This article reviews the roles, potential, and challenges of using MNPs in advancing cancer immunotherapy. The application of MNPs in boosting immunotherapy, and its perspective role in research and development is also discussed.

## Introduction

Cancer is a leading cause of death worldwide and has been a global public health issue for centuries. Although the incidence of some cancers has started to decline as a result of the better prevention of the associated risk factors and screening at the early preneoplastic stage, the incidence of some cancers, e.g., the malignancy of lung, breast, prostate, and corpus uteri, remains increasing [1]. It is projected that approximately 2 million new cases and 600,000 deaths will be reached in the USA at the end of 2023. This results in unevaluable social and economic impacts. The investigation for curative cancer treatments has, thus, been a hope for improving not only the quality of life for patients but also the holistic aspects of society.

Many therapeutic modalities are developed to improve the outcome of cancer treatments. One of the emerging tools for cancer treatment is immunotherapy [2]. Since the ability to evade immune surveillance is one of the cancer hallmarks [3, 4], harnessing endogenous immunity or manipulating immune activation by exogenous factors has become very promising in recent years. Strategies to manipulate the self-immunity of patients to turn against cancer cells are investigated, ranging from the development of monoclonal antibodies against specific oncoproteins [5] to the modulation of innate and adaptive immune cells [6, 7]. Cancer immunotherapy has revolutionized cancer treatment both in solid cancer and hematologic malignancies for years and is a breakthrough in the oncotherapeutic field [8]. Various cancer types have positive clinical responses to immunotherapy, such as metastatic melanoma [9], advanced renal cell carcinoma [10, 11], advanced or metastatic triple-negative breast cancer [12], metastatic non-small cell lung cancer [13, 14], and resectable lung cancer [15, 16]. Nowadays, multiple immunotherapy-based treatments have been approved by the USA Food and Drug Administration (FDA), including monoclonal antibodies [9, 17], immune checkpoint blockades [17], and chimeric antigen receptor (CAR) T cell products [18]. Some medications are in ongoing clinical trials and hold a high promise of being approved a few years later.

Cancer immunotherapy is such a breakthrough therapeutic modality that it is an exciting and promising leap toward cancer curation. It is acknowledged that there remain some challenges that need improvement for this method [2, 19]. Examples include that immunotherapy has only had satisfactory outcomes with specific subtypes of cancer. These include a defined tumor with the immune microenvironment, the so-called “hot” tumors. Moreover, the immune cells that are involved in cancer cell eradication can become exhausted when exposed to modulation by cancer and other stromal cells in the tumor microenvironments (TMEs) [20, 21]. Many studies are thereby investigating novel methods to overcome these civil wars with the endogenous enemies [8]. Among the emerging technologies to boost cancer immunotherapy, nanomedicine is one of the tools that has been applied and has become impactful for decades [22]. One of the emerging in immunotherapy is magnetic nanoparticles, especially magnetite nanoparticles (MNPs, Fe_3_O_4_) [23]. Safety windows have long been used when MNPs have been used in the medical field. MNPs are friendly tools for biomedical investigators, and the studies on this topic are increasing worldwide [24]. In this narrative review, PubMed, Science Direct, and Scopus databases were searched and the pertinent original and review articles are included in the narration and discussion. The advancement of MNPs application in cancer immunotherapy is reviewed, and the state-of-the-art and the challenge, along with the development of MNPs for different immunological aspects of cancer immunotherapy, are also discussed.

## Challenges in cancer immunotherapy and the assistance of nanomedicine

With a notable success rate of cancer immunotherapy, it is important that this therapeutic modality remains subject to ongoing refinement and advancement. Many challenges arise along with the translation to patients and at the research and development sections [19]. For instance, non-specific immune stimulation often precipitates pronounced inflammatory responses and may cause undesired or severe side effects [23]. For example, checkpoint inhibitor therapy, while liberating immune responses from inhibitory constraints, can inadvertently trigger uncontrolled reactions. This may substantially impact the patient’s physiological milieu, giving rise to unintended immune-related adverse events [25]. In addition to these challenges, it is crucial to recognize that the clinical efficacy of immunotherapeutic approaches remains confined to a select subset of patients, contingent upon the inherent immunogenicity of distinct tumor types, particularly within the ambit of immuno-oncological strategies aimed at stimulating specific anti-tumor immune responses [26].

Nanoparticles are various classes of matter sized in a nanoscale ranging from 1–100 nm. These ultrafine particles can be synthesized from various materials and thus result in different properties that can be used in multi-purpose applications [27]. One emerging nanomaterial in medical applications, including cancer immunotherapy, is MNPs, especially the aforementioned MNPs [23]. MNPs have garnered approval from the USA FDA for applications in medical diagnostics, including magnetic resonance imaging (MRI) and iron replacement therapy [28, 29]. Furthermore, the European Medicines Agency (EMA) has approved the use of this iron oxide particle, exemplified by NanoTherm^®^, to treat recurrent glioblastoma multiforme [30, 31]. MNPs have also emerged as an auspicious platform for drug delivery systems [32, 33]. These distinctive features render MNPs amenable to theragnostic applications and have prompted extensive exploration of their potential utility in innovative oncotherapeutic interventions.

## MNPs: from a safe diagnostic to therapeutic tools in oncology

Magnetite, an iron oxide mineral (Fe_3_O_4_), is abundantly present in natural environments. It exhibits an inverse spinel crystalline structure [34]. The oxygen ions closely pack face-centered cubic lattices, and ions with iron ions are located in the interstitial positions between these oxygen ions. These interstitial spaces comprise two different types: tetrahedral and octahedral sites. Magnetite contains Fe^2+^ ions in the octahedral sites (Fe^2+^_Oh_), while both Fe^3+^ ions occupy separated octahedral sites (Fe^3+^_Oh_) and tetrahedral sites (Fe^3+^_Td_) in a 1:1:1 ratio. The chemical composition is denoted as Fe^3+^_Td_(Fe^3+^Fe^2+^)_Oh_O_4_^2−^. Furthermore, under a Curie temperature range of 580°–600°C, the unit cell edge length of magnetite measures a = 8.408 Å [35]. MNPs exhibit electrical conductivity, optic, and magnetic properties, as documented in prior studies [36, 37]. Their broad applicability, the modulation of surface reactivity stands as a crucial focal point [38]. Given the diverse array of applications and interactions with various ions, biomolecules, or polymers, the precise management of surface reactivity concerning magnetite remains a subject of significance [39–43]. For example, MNPs have been useful for drug delivery, cancer therapy [44–46], and MRI [47]. Therefore, all these properties of MNPs have extensive utility in cancer therapy. Specifically, the encapsulation of pharmaceutical agents within MNPs or their surface attachment enables the precise localization of drugs to desired *in vivo* sites through the application of an external magnetic field [48–50]. This strategic design holds significant promise in targeted cancer treatment methodologies [51].

A variety of MNPs have garnered regulatory approval from the USA FDA for clinical deployment, i.e., ferumoxide [52], ferumoxtran-10 [53], ferumoxsil [54], ferucarbotran [55], ferumoxytol [56], and magtrace [57]. These MNPs have primarily been endorsed for the utilization within clinical imaging. Nevertheless, the relentless pursuit of optimized MNPs to enhance imaging efficiency is an ongoing investigation. Certain types of this nanoparticulate cohort, however, exhibit an unfavorable performance-to-cost ratio, while the others have been discontinued from clinical practice due to unmet efficacy expectations. In contrast, some modified MNPs show a particular advantage for clinical imaging, especially tumor visualization, based on the biological background of the diseases. A notable investigation by Bai et al. [58] showed the prospect of MNPs augmentation by incorporating a tumor-targeting peptide, cyclic arginine-glycine-aspartic acid (cRGD) peptide. This peptide is designed to selectively target the integrin alphavbeta3 (αvβ3) receptors, frequently overexpressed by the endothelial cells within angiogenic tumor vasculature. Such molecular modification facilitates the expedited accumulation of MNPs within murine tumor models, thereby significantly amplifying the efficacy of the MRI. Furthermore, MNPs have demonstrated remarkable versatility by integrating with supplementary imaging modalities, thereby conferring the potential for multi-modal imaging approaches [58]. An illustrative example is reported by Li et al. [59], in which the authors ingeniously embedded MNPs within nano micelles constituted of 1,2-distearoyl-sn-glycero-3-phosphoethanolamine-*N*-amino(polyethylene glycol)-5,000 (DSPE-PEG5k). These nano-micelles were conjugated with a near-infrared fluorescence dye [cyanine 5 (Cy5)] in conjunction with the tumor-targeting peptide bombesin, strategically designed to target G protein-coupled receptors overexpressed in diverse malignancies. This intricate construct precisely targets triple-negative breast cancer, MDA-MB-231, cells by the nano-micelles. Importantly, this innovative approach enabled dual imaging modalities, encompassing MRI by utilizing the inner MNPs component and near-infrared fluorescence imaging employing the Cy5 dye [59]. These findings underscore the inherent advantages of facile surface modification and functionalization of MNPs, which hold considerable promise, particularly within immuno-oncotherapeutic interventions.

## MNPs as an adjunctive treatment for cancer immunotherapy

### Modulation of macrophage activities by MNPs

Macrophages represent the frontline of the body’s defense mechanism against both infections and malignancies, playing a pivotal role in the innate immune system [60]. These versatile immune cells continuously survey tissues, identifying and eliminating senescent, abnormal, and deceased cells. Subsequently, they initiate an inflammatory process and activate effector cells to eliminate and digest pathogens and tumor cells effectively. The functional activity of macrophages is profoundly influenced by their tissue of residence, developmental origin, and the specific microenvironmental cues they encounter [61]. One intriguing aspect of macrophage behavior in the context of cancer is their role as tumor-associated macrophages (TAMs) [7]. These TAMs possess the unique ability to reactivate primed T cells within the TME by cross-presenting tumor antigens, a function that traditionally occurs in the draining lymph nodes. This dual role in both innate and adaptive immunity underscores their significance in anti-tumor responses [62]. While it is known that TAMs may not be as efficient as dendritic cells (DCs) in antigen presentation, they still possess this capacity. Furthermore, TAMs exhibit an ideal profile for acting as tumor surveillance through various mechanisms, including the secretion of cytokines such as interferon-gamma (IFNγ) and tumor necrosis factor-alpha (TNFα), the production of inducible nitric oxide synthase, and the induction of anti-tumor inflammatory response [63]. The tumor cells, however, turn against the immune systems and evade the phagocytosis by these immune cells by overexpressing “do-not-eat-me” signals [64]. By interacting with the inhibitory signals from tumor cells, the immune cells can become exhausted, and the phagocytotic and antigen-presenting functions could be compromised. The modulatory signals and cytokines from tumor cells can also alter the polarity of TAMs from the anti-tumorigenic M1 to the pro-tumorigenic M2-like phenotypes. The M2-like TAMs further diminish the anti-tumor potential of M1-like TAMs, thereby promoting tumor growth, immune evasion, and metastasis [65]. Consequently, extensive research efforts have been undertaken to enhance phagocytic activity and promote M1 polarization of TAMs through the utilization of MNPs as a promising approach.

There are several studies demonstrating that MNPs are helpful for a re-polarization of M2-like macrophages in TME to anti-tumorigenic M1-like macrophages. The *in vitro* study of leukemic cell lines showed that ferumoxytol, an FDA-approved MNP, can upregulate the markers of the M1 phenotype while downregulating the markers of the M2 phenotype of TAMs [66]. Ferumoxytol-treated mice also show increased polarization toward M1 phenotypes and increased infiltration of M1-like TAMs in a murine breast cancer model. *In vitro*, the study of mouse cancer cells Hepa 1-6 conducted by Zhang et al. [67] also showed similar trends. Dimercaptosuccinic acid (DMSA)-coated MNPs induced proliferation, migration, and chemotaxis of mouse macrophage RAW264.7 cells, to directly kill Hepa 1-6 cells [67]. In breast cancer mouse models, a biomimetic polymer magnetic nanocarrier poly lactic-co-glycolic acid (PLGA)-ion-R837@M (PIR@M) selectively targeted and polarized TAMs, resulting in the upregulation of both the pro-inflammatory cytokines and the infiltration of T-lymphocytes in tumor tissues [68]. Rao et al. [69] demonstrated the effects of genetically engineered cell-membrane-coated magnetic nanoparticles (gCM-MNs) on malignant melanoma and triple-negative breast carcinoma mouse models and found that the gCM-MNs facilitated the macrophage phagocytosis of cancer cells. Also, they triggered anti-tumor immunity of T cells and significantly prolonged the overall survival of the mice by controlling both local growth and distant metastasis.

**Figure 1:**
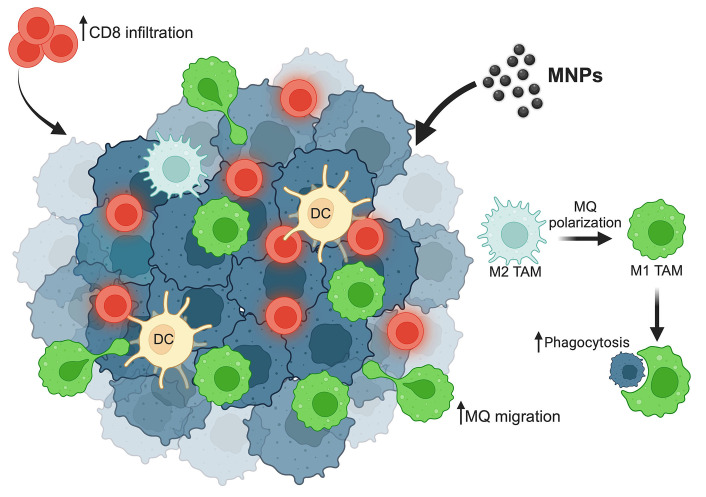
Summary of MNPs efficacy on macrophages’ activities. MNPs enhance macrophage repolarization from the M2 type into the M1 type, which plays a role in cancer cell phagocytosis. MNPs increase macrophage as well as cytotoxic CD8 T cell migration to the tumor site. ↑: increasing; MQ: macrophage. This figure was created by BioRender.com

In addition, MNPs can also enhance the efficacy of immunotherapeutic drug delivery to tumors by the guidance of an external magnetic field. By this application, the modified MNPs, show the effectiveness of carrying the monoclonal antibody against cluster of differentiation 47 (CD47), a do-not-eat-me signal, expressed on cancer cells to prevent the inhibitory interaction with the signal-regulatory protein alpha (SIRPα) on macrophages [69]. CD47 is also expressed on the surface of normal cells to protect them from the phagocytosis of macrophages. The magnetic field guidance for MNPs delivery is, thereby, also beneficial for preventing the reverse reaction of cancer immunotherapy. Although the application of MNPs to enhance macrophages’ activity is promising, the number of studies is limited. Further investigation will thus be needed for clinical translation. The summary of MNPs modulating macrophage activities is demonstrated in Figure 1.

### Turning “cold” to “hot” tumor by MNPs

Tumor-promoting inflammation is defined as one of the cancer hallmarks in which the immune cells play crucial roles in this process [3, 4]. Various immune cells are populated in the TMEs and may play different roles, both anti-tumorigenic and pro-tumorigenic effects. The immunological state of the TMEs could be classified as “cold” or “hot” regarding the infiltration of leukocytes from blood circulation to the tumor site [70]. The “cold” tumor usually has less cytotoxic T lymphocytes (CTLs) infiltration. In contrast, the infiltration of immunosuppressive cells is remarkable, such as regulatory T cells (Tregs), myeloid-derived suppressor cells (MDSCs), and M2-TAMs. These “cold” TMEs can compromise the functions of infiltrated CTLs, resulting in immune exhaustion. In line with these effects, cancer immunotherapy is, therefore, less effective for “cold” tumors. Induction of the “cold” TMEs toward a “hot” tumor is then the strategy to enhance the effectiveness of cancer immunotherapy [71].

A “hot” TME of tumors is significantly built up and infiltrated by natural killer (NK) cells, DCs, and CTLs that are not yet exhausted [71]. This condition can prime the therapeutic effects of the administered cancer immunotherapy. Several reports show that MNPs can enhance the functions of these “hot” populations of the TMEs, both *in vitro* and *in vivo*. Jiang et al. [72, 73] found that ZnCoFe_2_O_4_@ZnMnFe_2_O_4_ MNPs lead to the activation of interleukin-2 expression and the activation of NK cells to infiltrate the tumor. Fe_3_O_4_@SiO_2_ MNPs coated NK cells also increase the expression of cytokines and receptors compared to unstimulated NK cells. Ranges of *in vitro* studies also demonstrated the preservation of NK cell function by attachment with MNPs in different models and might be beneficial for immunotherapy.

**Figure 2:**
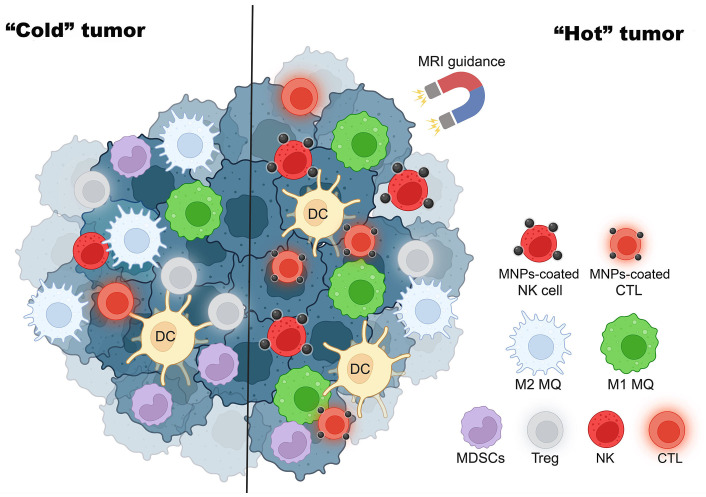
MNPs turn “cold” tumors into “hot” tumors. MRI guidance increases MNP-coated effector immune cells such as NK cells and CTLs to tumor sites and enhances cancer cytotoxicity. This figure was created by BioRender.com

Apart from the enhancement of immune cell functions, MNPs can also be applied for the induction of “hot” immune cells to the tumor. CTLs can be attracted by external magnetic force by loading the cells with citrate-coated MNPs-citrate. The study of Sanz-Ortega et al. [74] indicates that CD8 T cells attached with 3-aminopropyl-triethoxysilane (APS)-coated MNPs can be magnetically recruited to the tumor by MRI guidance and shown to improve tumor infiltration. Meng et al. [75] also showed that MNPs-coated lung cancer membranes could extend the half-life of tripeptidyl peptidase 1 (TPP-1) and maintain the activation of CTLs to inhibit tumor growth. As shown in Figure 2, MNPs convert “cold” tumors into “hot” tumors.

### MNPs and hyperthermia therapy for activation of immunotherapy

Hyperthermia therapy or thermal therapy is a technique that increases the temperature at the tumor site for the objective of tumor ablation [76, 77]. However, hyperthermia alone provides limited efficacy for cancer treatment, so it is usually used to prime the treatment by other modalities, such as radiotherapy [78] or chemotherapy [79, 80], for synergistic effects. Hyperthermia therapy is relatively less invasive, has less mutagenic potential, fewer side effects, and increases tumor specificity [51]. There are multiple techniques for applying hyperthermia therapy, such as photothermal therapy (PTT) [81], photodynamic therapy (PDT) [82, 83], and magnetic hyperthermia (MHT) [51, 84].

MNPs can generate heat by converting the magnetic energy to thermal energy when exposed to the alteration of the magnetic field [51]. The heat generation process is called MHT, which could be applied for cancer treatment. MHT has better tissue penetration than PTT and PDT due to its alternating magnetic field, which is not limited by beam penetration to external skin from PTT, which uses lasers to generate heat. Hyperthermia therapy can destroy the tumor cells, thus releasing cytokines and chemicals that recruit immune cells to initiate a response and induce immunogenic cell death [85–87]. This process can expose the tumor-associated neoantigen and danger-associated molecular patterns (DAMPs) of cancer cells to recruit and prime CTLs. However, MHT therapy alone is often insufficient to induce complete regression of poorly immunogenic tumors in an immunosuppressive TME. Therefore, other immunological tools are needed, not only turning the tumor to be “hot” but also functioning as a cytotoxic agent for tumor cell destruction [88, 89].

As for the future, it is agreed amongst the studies that MHT-immunotherapy, specifically by the MNPs, has safe and efficient synergistic therapeutic effects against cancer. The harmony between PTT and MHT, when integrated with immunotherapy, can serve as a multifunctional therapeutic platform to inhibit tumor growth, metastasis, and recurrence [89–91]. The combined therapies not only serve its antitumor outcomes but also act as a potent immunological stimulant, indicating a promising area of research for the future.

## MNPs enhancing the effects of immune checkpoint blockade

Immune checkpoints have a vital role in immunological homeostasis for balancing the effects of CTLs in eradicating the damaged cells and recognizing the normal cells. The program cell death protein-1 (PD-1) expressed on T cells can bind with its ligand, program cell death protein ligand-1 (PD-L1), on normal cells to signal the information of self-antigen and prevent the attack by CTLs [92, 93]. Cancer cells, however, hi-jack and overexpress the PD-L1 on their cell surface, leading to the evasion of cytotoxicity caused by CTLs. The immune checkpoint blockades, namely anti-PD-1/PD-L1 and anti-cytotoxic T-lymphocyte-associated protein 4 (CTLA4), have thus been developed to restore the functions of CTLs. However, the immune checkpoint blockades are shown to have a limited overall response rate with frequently associated unwanted immune-related adverse events [93, 94]. This limitation limits clinical application and needs further investigation to improve clinical outcomes with minimal adverse effects.

**Figure 3:**
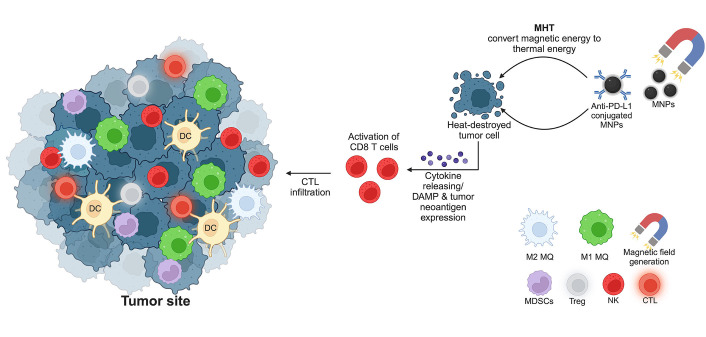
MNPs and hyperthermia therapy for cancer treatment. The generation of a magnetic field initiates a conversion of magnetic energy to thermal energy, resulting in the destruction of tumor cells by heat. Consequently, CD8 T cells are primed by the expression of DAMPs and tumor-associated neoantigen. The release of cytokines attracts more CTLs to the tumor site to kill cancer cells. MQ: macrophage. This figure was created by BioRender.com

As aforementioned, MHT generated by MNPs can recruit and prime the activation of CTLs by exposing tumor neoantigen and DAMPs. The combination of MNPs and the immune checkpoint blockades is thus promising to enhance the anti-tumor activity of the infiltrated CTLs [95, 96]. Conjugating the immune checkpoint inhibitors and MNPs significantly enhances tumor targeting and therapeutic efficacy. Moreover, conjugation of anti-PD-L1 and CTLs activators (CD3 and CD28) to the fucoidan-dextran-coated MNPs can improve the tumor accumulation of CTLs by magnetic guidance [97]. This combination improves the survival of tumor-bearing mice and minimizes the side effects due to a lower therapeutic dosage. With the success of this application, conjugating MNPs with various antibodies targeting cancer cells is a promising method to improve the outcome of cancer immunotherapy, especially for those that can synergize the effects of MHT [98]. Although highly promising for clinical translation, the information on the synergistic effects of immune checkpoint blockades and MNPs remains limited, especially in the clinical setting. A schematic summary of MNPs and hyperthermia therapy for activation of immunotherapy is depicted in Figure 3.

## Concerning adverse reactions from the MNPs in clinical use

The use of MNPs in cancer immunotherapy, like other nanoparticles, may pose some undesirable side effects. MNPs might exhibit toxicity due to their composition or interaction with biological systems, potentially causing cellular stress or damage not only to cancer but also to normal cells [99, 100]. A report also shows that MNPs might have an immunogenic property and then trigger immune reactions, leading to inflammation or immune responses against the nanoparticles themselves, impacting their efficacy or causing adverse reactions [101]. Using MNPs in the *in vivo* systems shows a risk of accumulating in organs, potentially causing localized toxicity or interfering with normal organ function [102]. MNPs might persist in the body over extended periods, raising concerns about potential long-term health effects or accumulation-related issues [103]. For hyperthermia-based therapy, the application of external magnetic fields might pose risks, such as tissue heating or adverse effects if not carefully controlled [46]. To cope with these concerns and possible issues, scientific efforts are focused on refining nanoparticle design, regulating dosages, and conducting comprehensive safety evaluations in both preclinical and clinical settings to mitigate these concerns. It is imperative to rigorously monitor and persistently investigate potential side effects linked to the utilization of MNPs in cancer immunotherapy for effective understanding and management.

## Current status of translating MNPs to clinical studies

Research involving MNPs in boosting immunotherapy for clinical studies has been ongoing but is still primarily in the preclinical stages. Their translation to clinical studies was progressing cautiously due to the need for further understanding of their safety profiles, optimal dosages, and potential long-term effects in humans for the purpose of treatment. Researchers are exploring the feasibility of using MNPs to enhance various aspects of immunotherapy, such as improving the delivery of immune checkpoint inhibitors, vaccines, or other immunomodulatory agents to tumor sites. Some studies are investigating the combination of MNPs with other therapeutic modalities, aiming to maximize their potential in cancer treatment [23, 104].

Several clinical studies demonstrated that MNPs are safe for use in patients [105]. Up to the present, MNPs have been mainly studied in clinical trials as a tool for improving diagnosis, e.g., to visualize cancer metastasis [106, 107] or to suggest the probability of benign *versus* malignant lymph nodes in pediatric patients [108]. Morbidity and quality of life after receiving MNPs in cancer patients were also evaluated [107]. The applications of MNPs for cancer treatment at the clinical trial levels are mostly used for hyperthermia treatments, as studied in glioblastoma [109] and bone metastasis cancer [110]. However, as many preclinical studies suggest that magnetic-induced hyperthermia can activate the immune cells in the TMEs to inhibit the tumor progression, the effects that resulted in the clinical trials for MNP hyperthermia could not be totally excluded from the immunological consequences. Up to the current search for this review, the clinical trial report on the success of using MNPs for immunotherapy has not been available. It is also predicted that a few studies could be moving to clinical trials in the next few years [111].

## Conclusions

**Figure 4:**
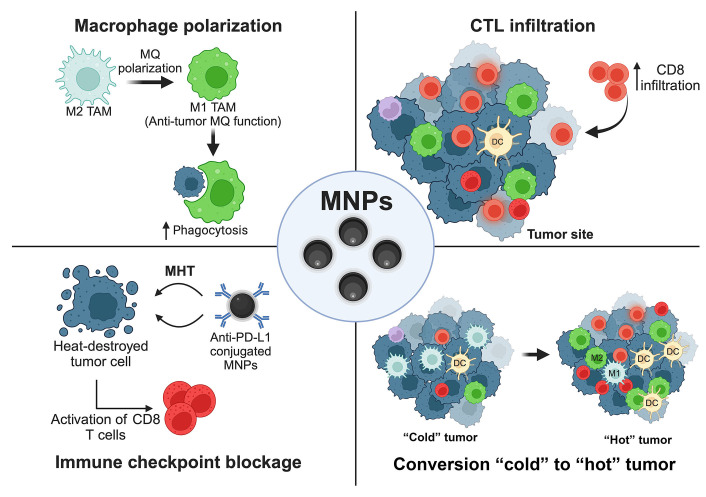
Schematic summary. MNPs (Fe_3_O_4_) provide an improvement for cancer immunotherapy. ↑: increasing; MQ: macrophage. This figure was created by BioRender.com

Many preclinical studies demonstrated the efficiency of using MNPs to boost the effects of immunotherapy by several strategies, i.e., modulating the functions of leukocytes, providing the appropriate TMEs for CTLs, and combining with monoclonal antibody-based medication as depicted in the schematic summary (Figure 4) [23].

The applications of MNPs for imaging, drug delivery, and hyperthermia treatment are currently in many clinical trials. Nevertheless, clinical studies for MNPs as an adjunctive modality of cancer immunotherapy are still lacking and thus limit the translation to patients at the present time. The adverse effects of using MNPs remain a concern and need more investigation, especially pharmacokinetics and pharmacodynamics when MNPs are combined with other strategies, such as hyperthermia. Therefore, more clinical investigations are urgently needed to ensure the efficacy of the booster effects of MNPs on cancer immunotherapy and the safety issues after the combination. The direction in the next few years should then address these issues and might start with the surveys of immunological surrogate outcomes derived from the previous clinical trials that use MNPs for other purposes rather than immunological boosting. This might help move forward the research on MNPs for improving cancer immunotherapy in the near future.

## Abbreviations

CTLs: cytotoxic T lymphocytes
DAMPs: danger-associated molecular patterns
DCs: dendritic cells
FDA: Food and Drug Administration
MDSCs: myeloid-derived suppressor cells
MHT: magnetic hyperthermia
MNPs: magnetite nanoparticles
MRI: magnetic resonance imaging
NK: natural killer
PD-L1: program cell death protein ligand-1
PTT: photothermal therapy
TAMs: tumor-associated macrophages
TMEs: tumor microenvironments
Tregs: regulatory T cells

## Declarations

### Copyright

© The Author(s) 2024.
